# Supplementary material for: The Effects of Postmortem Time on Muscle Trout Biochemical Composition and Structure
Source: Foods. 2023 May 11;12(10):1957. doi: 10.3390/foods12101957 (PMC10217499; doi:10.3390/foods12101957)
Supplement: Supplementary file 1 [file foods-12-01957-s001.zip › foods-2362014-supplementary.pdf]

Supplementary data

The effects of postmortem time on muscle trout biochemical composition and structure

Arno Germond[a*], Annie Vénien[a], Christine Ravel[a], Brayan Castulovich[a], Jacques Rouel[a], Morgane Hutin[a], Sara Mezelli[a], Sandy Lefin[a], Pierre-Sylvain Mirade[a], Thierry Astruc[a].

[a]UR370, QuaPA, INRAE, 63122 Saint-Genès-Champanelle, France

*Corresponding authors.

Email: arnaud.germond@inrae.fr

Supplementary Table S1. Molecular bonds identified from the classification model of fresh muscles at D0 against D15.

Wavenumber	Biomarker for	Molecular bond	Biological compounds
1002 cm^{-1}	D0	Phe, Tyr	Aromatic compound, protein
1018 cm^{-1}	D15	CO, CC, OCH	Carbohydrates, polysaccharides
1186 cm^{-1}	D0	Amide III	Amide III
1172–1176 cm^{-1}	D0	CC of DNA, PO_2 stretching	DNA, PO_2, nucleic acids, carbohydrates
1247 cm^{-1}	D0	PO_2	nucleic acids (RNA)
1363 cm^{-1}	D15	CH_2, CC	Polysaccharides
1469 cm^{-1}	D0	CH_2 bending of lipids	Saturated lipids

1490 cm^{-1}	D15	CC, CH	amide I, fatty acids
1553 cm^{-1}	D0	Amide II	Predominantly the α-helix of amide II
1635 cm^{-1}	D0	β-sheet structures of amide I	Proportion of secondary protein structures (shoulder peak of amide I)
1670 cm^{-1}	D15	Antiparallel β-sheet structures of amide I	Antiparallel β-sheets, fatty acids (shoulder peak of amide I)

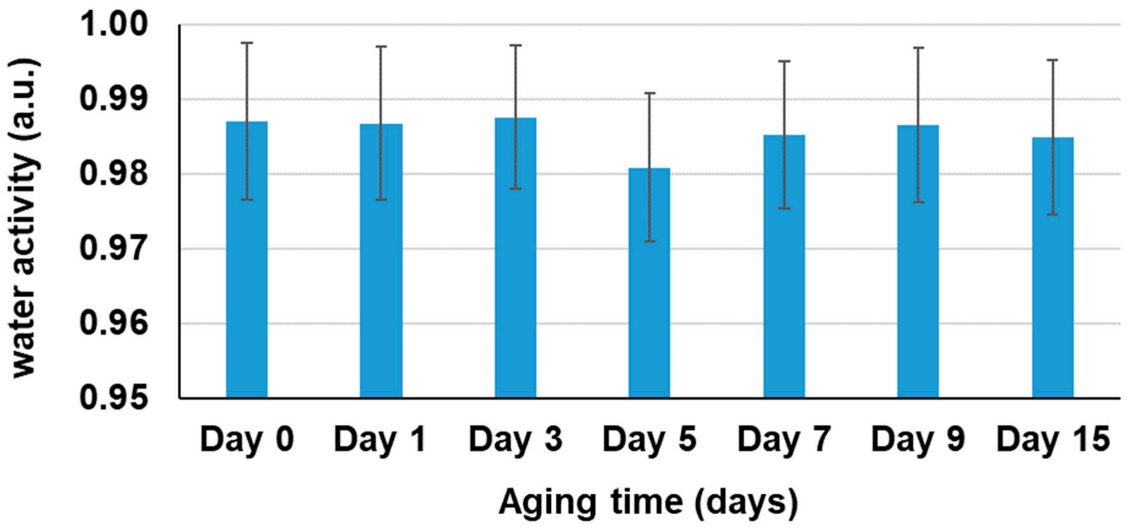

Supplementary Figure S1. Water activity (a_w) averaged across 6 fish (n=6). Error bar shows standard deviation.

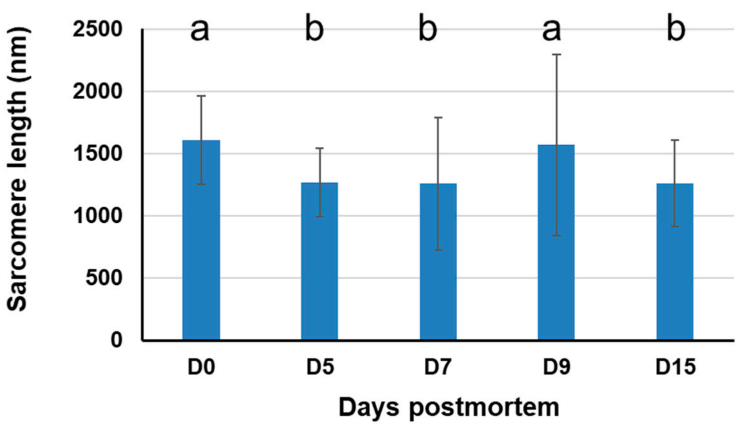

Supplementary Figure S2. Evolution of sarcomere length *postmortem*. Days with the same letter suggest that the average sarcomere lengths are not significantly different according to an ANOVA followed by a post-hoc Tukey HSD test.

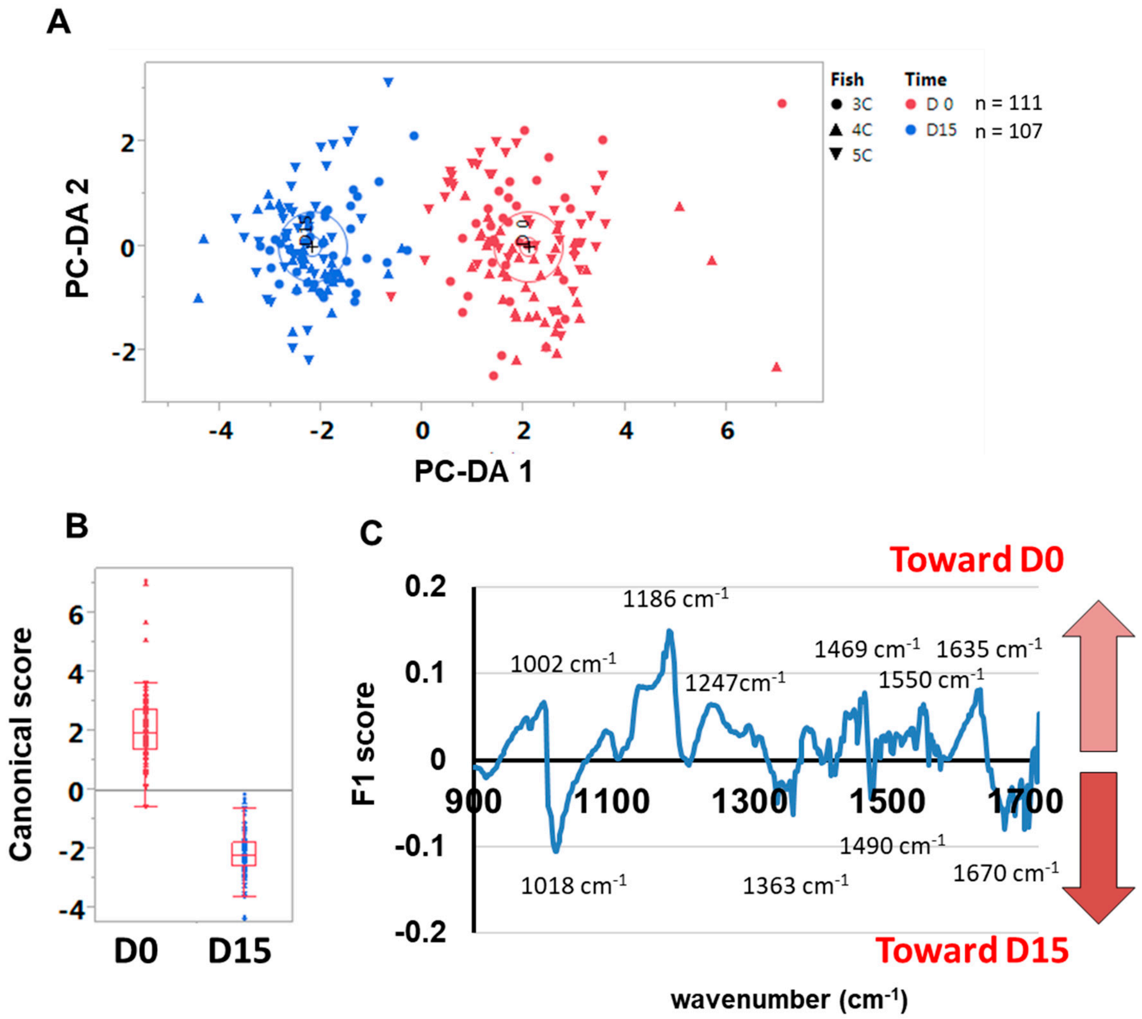

Supplementary Figure S3. Molecular bonds identified from the classification model of fresh muscles (D0) against D15. The associated biological compounds were identified from the literature.

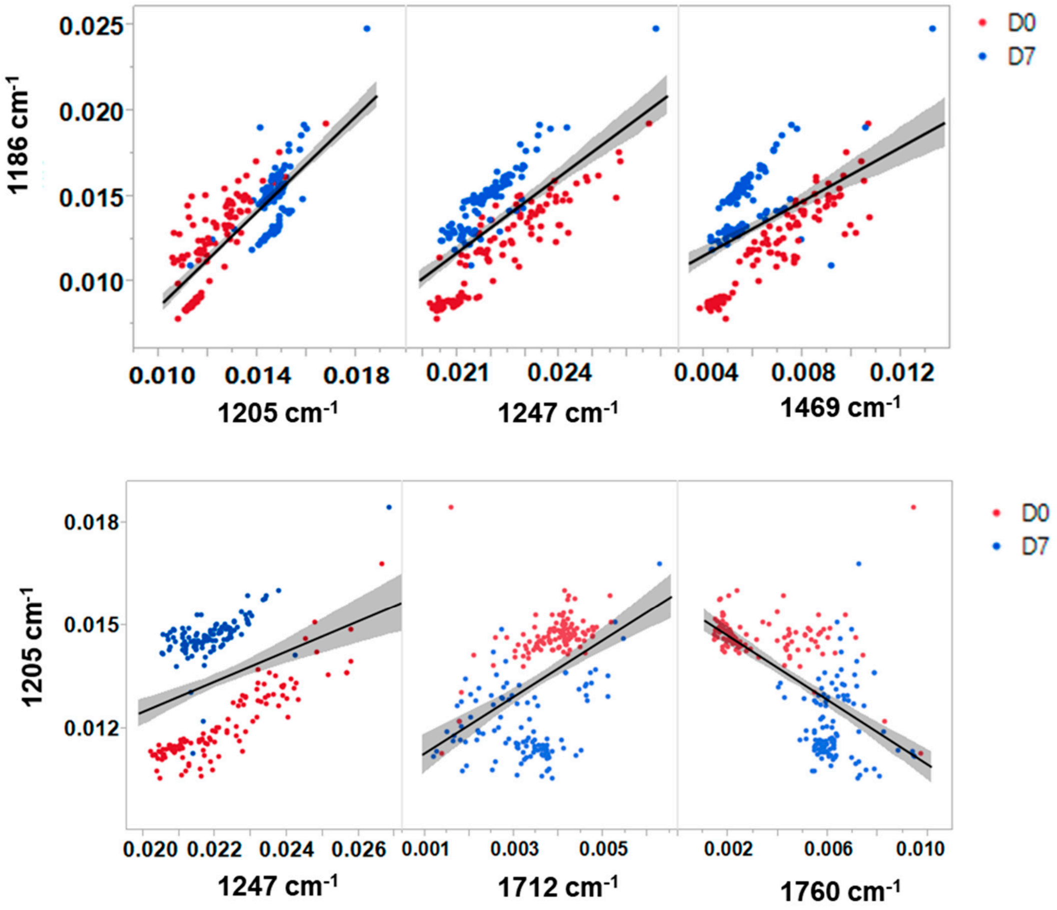

Supplementary Figure S4. Spectral signal intensities between pairs of major peaks. Spectral intensities were compared for several pairs of wavelengths, at D0 and D7, indicating different tissue compositions depending on the freshness.
